# Primate-specific Long Non-coding RNAs and MicroRNAs

**DOI:** 10.1016/j.gpb.2017.04.002

**Published:** 2017-06-08

**Authors:** Hassaan Mehboob Awan, Abdullah Shah, Farooq Rashid, Ge Shan

**Affiliations:** CAS Key Laboratory of Innate Immunity and Chronic Disease, CAS Center for Excellence in Molecular Cell Science, School of Life Sciences, University of Science and Technology of China, Hefei 230027, China

**Keywords:** Non-coding RNA, Primate-specific, LncRNA, MicroRNA, Evolution

## Abstract

**Non-coding RNAs** (ncRNAs) are critical regulators of gene expression in essentially all life forms. Long ncRNAs (**lncRNAs**) and **microRNAs** (miRNAs) are two important RNA classes possessing regulatory functions. Up to date, many **primate-specific** ncRNAs have been identified and investigated. Their expression specificity to primate lineage suggests primate-specific roles. It is thus critical to elucidate the biological significance of primate or even human-specific ncRNAs, and to develop potential ncRNA-based therapeutics. Here, we have summarized the studies regarding regulatory roles of some key primate-specific lncRNAs and miRNAs.

## Introduction

RNA is believed to be the precursor to all the current life forms on earth. The RNA World, first posited in late 1960s, proposes that self-replicating RNA molecules initially contained both the information and the function needed for their perpetuation before the emergence of the first cell [1]. Over the course of time, information contained in RNA had been passed on to the more stable DNA, while most catalytic functions were delegated to more versatile proteins, thereby limiting the role of RNA as an intermediate between genes and proteins [2]. The human genome contains approximately twenty thousand protein-coding genes [3], which make up to 2% of the entire genomic sequence, whereas more than 80% of the genome is pervasively transcribed [4]. Pervasive transcription of human genome is in stark contrast to the small portion of transcription of protein-coding genes. Given their abundance, most of these so-called non-coding RNAs (ncRNAs), which are not translated into proteins, were previously believed to be spurious transcriptional noises arising due to low fidelity of the RNA polymerase (RNAP) [5]. However, accumulating findings have demonstrated that a lot of these ncRNAs play vital roles in many life events [6], [7], [8], [9], [10].

ncRNAs can generally be classified into either housekeeping or regulatory ncRNAs. The housekeeping ncRNAs are constitutively expressed, which include tRNAs, rRNAs, small nuclear RNAs (snRNAs), and small nucleolar RNAs (snoRNAs). Regulatory ncRNAs, on the other hand, can further be classified into long ncRNAs (lncRNAs) and small ncRNAs based on the transcript length. The former has transcript length of greater than 200 bp, whereas the latter has transcript length of less than 200 bp [11].

lncRNAs can be placed into six categories on the basis of their proximity to the protein-coding genes in the genome. An lncRNA is categorized as sense or antisense if the sequence of the lncRNA overlaps the sense or antisense strand of a protein-coding gene, respectively. If the sequence of the lncRNA is present on the opposite strand of a nearby protein-coding gene and transcription of both are initiated in close genic proximity, then the lncRNA transcripts are called divergent lncRNAs. Intronic lncRNAs are derived entirely from within the intron of another transcript. They can be classified as sense-overlapping or antisense if the lncRNA gene lies within the intron of coding gene on the same strand or on the opposite strand, respectively [11]. These lncRNAs can either be a product of pre-mRNA processing or the result of an independent transcription [11], [12]. Intergenic lncRNAs (lincRNAs), on the other hand, are located in the genomic interval between two protein-coding genes [12].

Small ncRNAs are very diverse and include microRNAs (miRNAs), *P*-element-induced wimpy testis-interacting RNAs (piRNAs), and small interfering RNAs (siRNAs) [13], [14], [15], [16]. miRNAs are the most studied small ncRNAs, representing approximately 4% of the genes in human genome and regulating more than one third of the expressed genes post transcriptionally [17]. Roughly half of the miRNA genes are present in the intergenic regions under control of their own promoters or shared promoters in case of poly-cistronic microRNA clusters [18]. The remaining miRNA genes are located within protein-coding gene, where they are under the influence of host-gene promoters or miRNA-specific promoters [18], [19]. RNAPII-transcribed miRNA transcript undergo subsequent processing in nucleus and cytoplasm to give rise to the mature miRNA [19], [20], which gets incorporated into the RNA-induced silencing complex (RISC). By means of partially or fully complementary base-pairing between 5′ seed sequences of miRNAs and target mRNAs, miRNAs repress protein translation and/or induce mRNA decay [21]. The conservation of seed region is important in grouping miRNAs into families [22], [23], [24].

Undoubtedly, RNAs are constantly evolving, starting from the RNA world. New ncRNAs have also been emerging in primates after their separation from other mammals [25]. lncRNAs and miRNAs are the most analyzed ncRNAs from the evolutionary view, and a handful of primate-specific lncRNAs and miRNAs have been studied for their lineage-specific roles.

## Evolution of lncRNAs

As it has been observed that majority of the lncRNAs exhibit low sequence conservation, they may exhibit different pattern of conservation in contrast to protein-coding genes. While protein-coding genes must preserve the ORF in order to retain the function, lncRNAs on the other hand maintain their functionality by means of preserving their short sequence stretches or structural motifs that may serve as functional domains [26]. The conservation of lncRNAs can be at the sequence, genomic synteny, functional, or structural level. There are various mechanisms by which lncRNAs may have originated. The first such mechanism is the gradual transformation of a protein-coding gene sequence into functional ncRNA sequence [12]. For instance, *Xist* encodes an lncRNA critical for the X-chromosome inactivation in eutherian mammals. Although well conserved in eutherians, *Xist* homolog is not found in other mammals [27]. Except the six exons containing tandem repeats, the promoter region and the remaining four out of ten exons of *Xist* gene have sequence homology with *Lnx3* that encodes the ligand of numb protein-X 3, a ubiquitin E3 ligase [28]. Evolution of *Xist* lncRNA was therefore thought to result partially from the loss of function of the protein-coding gene. This event occurred after the divergence between eutherians and marsupials, suggesting independent evolution of dosage compensation in both lineages [27].

Another possibility of lncRNA origin is chromosome rearrangement. Two untranscribed sequences that were previously well-separated juxtaposed each other, giving rise to an lncRNA. An example for this kind of lncRNA origin comes from the observation that a dog testis-derived lncRNA (supported by ESTs BM537447, C0597044, and DN744681) emerged in canid lineage approximately 80 million years ago after the last common ancestor of canids and bovids [12]. It is of note that this lncRNA locus spans two regions that are tens of megabases apart in other eutherian mammals [12], [29].

Except for a few extensively-studied lncRNAs, origin of most lncRNAs still remains vague. Duplication of lncRNA to give rise to a new lncRNA has also been proposed. For instance, mouse *nuclear enriched abundant transcript 2* (*Neat2*) [30] and mouse testis-derived lncRNA *AK019616* are paralogous to each other [28]. However, two independent studies in zebrafish and humans have shown that lncRNAs rarely have extensive sequence similarity outside of the shared repetitive elements [31], [32]. These observations suggest that lncRNA genes, instead of originating from duplication events, arise *de novo* from non-exonic sequence or from transposable elements (TEs) [33].

Another possible way by which a functional lncRNA could emerge is by the TE insertion. Owing to their ability to move and spread in the genome, as well as their potential to introduce regulatory sequences, TEs represent a major force in increasing the lncRNA repertoire [33]. Besides duplication events or insertion of TEs, new lncRNA genes can also originate through reuse of the general transcriptional context of other functional genes [34]. In one study, it is proposed that new lncRNA genes may originate by strengthening of the U1-polyadenylation signal (PAS) axis that can extend the transcriptional length of lncRNA genes present anti-sense to functional coding genes [35]. Presumably non-coding transcripts, either originating from pervasive transcription or through aforementioned mechanisms, have evolved functional benefits to the specific lineage. They would become functional lineage-specific lncRNAs such as primate-specific ones, which we are going to discuss below.

### *5S-OT*

**Figure 1:**
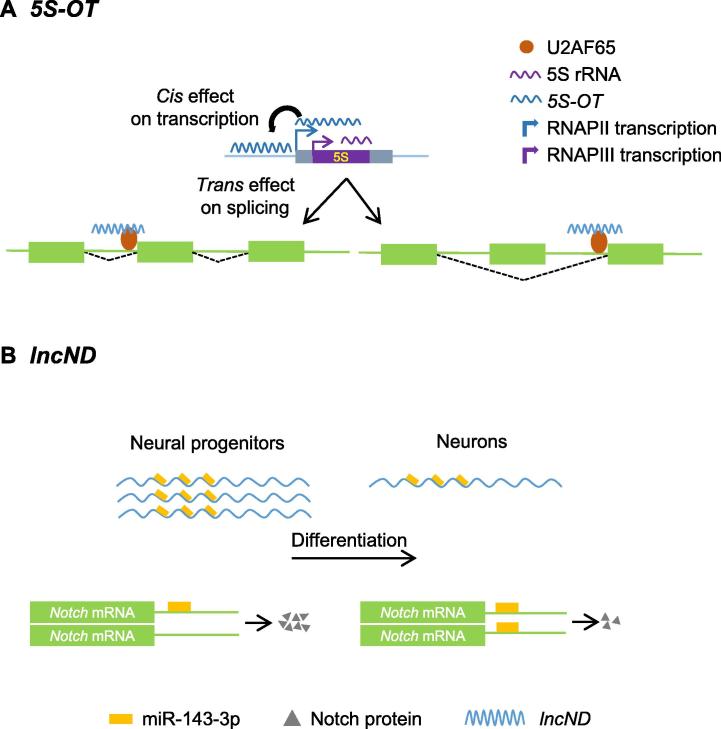
**Functional mechanisms of some primate-specific lncRNAs** **A.** In mammals, *5S-OT* regulates its own transcription by acting in *cis*, whereas in humans it can also act in *trans* by interacting with splicing factor U2AF65, thus modulating alternate splicing. **B.** In neural progenitors, *lncND* is highly expressed sequestering miR-143-3p, thereby indirectly increasing Notch protein expression necessary for maintenance of neural progenitors. During differentiation, expression of *lncND* decreases and free miR-143-3p represses *Notch* mRNA, indirectly decreasing Notch protein expression in support of the differentiation of progenitor cells. RNAP, RNA polymerase; U2AF65, U2 snRNA auxiliary factor 2 of 65 kDa.

Alu elements are short stretch of DNA highly abundant in the primate genome. There are more than one million copies of Alu elements dispersed throughout the human genome [36]. Belonging to short interspersed nuclear element (SINE) family, these primate-specific retro-transposable elements (REs) play a major role in shaping the evolution of the genome, thereby constantly regulating the repertoire of the regulatory elements. 5S rRNA is an ancient, highlyconserved ncRNA that is present in essentially all domains of life. Recently, we have described an lncRNA expressed from 5S rDNA locus and therefore named it *5S-OT* (OT stands for overlapping transcript) [33]. From fission yeast to humans, this lncRNA is relatively conserved, and regulates the transcription of 5S rRNA by RNAPIII in *cis* (Figure1A) [37], [38]. Conserved at the sequence, synteny, and functional level in eukaryotic cells, *5S-OT* may be one of the most ancient lncRNAs transcribed by RNAPII. In the anthropoidea suborder of primates including humans, however, insertion of an Alu element in the 5S rDNA locus leads to the generation of the *5S-OT* lncRNA harboring antisense Alu sequences at the 3′ end. The insertion also creates a polypyrimidine tract (Py) in the sequence of *5S-OT* lncRNA, which serves as the site for *5S-OT* to interact with splicing factor U2 snRNA auxiliary factor 2 of 65 kDa (U2AF65). *5S-OT* then brings U2AF65 to regulate alternative splicing of hundreds of human genes via Alu-mediated antisense:sense RNA−RNA pairing. This unique *trans* role in pre-mRNA splicing is primate-specific, as *5S-OT* of other mammals does not have Py site and thus does not interact with U2AF65. It has been shown in the study that *5S-OT* plays critical role in the differentiation of human cells. Since Alu elements are primate-specific, it is highly possible that this Alu and *5S-OT* dependent splicing regulation may have contributed to transcriptome divergence of primate from the rest of the mammals [37].

### *lncND*

Rani et al. have provided evidence for the involvement of a primate-specific lncRNA *lncND* (ND stands for neuronal development) in regulating an ancient signaling pathway controlling brain development [39]. Phylogenetic analysis shows that *lncND* is more conserved in Catarrhini (old world monkeys and apes) as compared to Platyrrhini (new world monkeys) [39]. A primate-specific sequence insertion occurred in the 5′ region of *lncND*. Further scrutinization of the sequence insert reveals the presence of 16 miRNA-recognition elements (MREs) for miR-143-3p. This suggests that *lncND* has sponging activity, thereby indirectly regulating the protein expression of miR-143-3p target genes. Among the neuro-developmental genes, *Notch-1* and *Notch-2* each contain a miR-143-3p MRE in the 3′UTR. High expression of *lncND* in neural progenitor cells as compared to neurons leads Rani and the colleagues to propose that *lncND* regulates the neuronal differentiation pathway by sequestering the miR-143-3p in early neurogenesis (Figure1B) [39].

### *lincHPAT5*

lincRNAs can be grouped according to the TEs residing in them, since lincRNA transcription takes place from sites frequently enriched with TEs [40]. Human endogenous retrovirus H (HERV-H) family of lincRNA has been linked to human embryonic stem cell (hESC) pluripotency [41], whereas HERV-V and HERV-K families are involved in the pre-implantation embryo development [42]. However, the role of individual lincRNA in pluripotency and embryogenesis is obscure. To answer this question, Durruthy-Durruthy et al. studied 23 human pluripotency-associated transcripts (HPATs) in hESCs [40]. Of the 23 transcripts examined, three transcripts, *HPAT2*, *HPAT3*, and *HPAT5*, showed elevated expression in the inner cell mass (ICM) with *HPAT5* being expressed at highest level. siRNA-mediated knockdown of all three HPATs in single blastomere of 2-cell stage human embryo blocked the blastomere from contributing to ICM of the developing embryo, suggesting essential role of these lincRNAs in embryonic development. CRISPR knockout of endogenous *HPAT5 (HPAT5-*KO*)* in hESCs demonstrated that *HPAT5* plays key roles in the pluripotency network. Sequence analysis revealed presence of MREs of let-7 family of miRNAs in *HPAT5*. The let-7 targeting of *HPAT5* is due to a point mutation occurring within Alu element of *HPAT5*, which generated let-7 seed sequence maybe in great apes 5–9 million years ago [40].

### *FMRR4*

There has been increasing interest in finding and characterizing lncRNAs with reference to their relevance to human disease. Fragile X syndrome (FXS) is a genetic disorder. A CGG triplet repeat occurs in the promoter of the gene encoding fragile X mental retardation gene 1 (*FMR1*) on X-chromosome, leading to the reduced or abolished expression of fragile X mental retardation protein (FMRP). A study in 2008 reported that a primate-specific lncRNA *FMR4* is located upstream of *FMR1*. *FMR4* likely shares a bidirectional promoter with *FMR1*. Knockdown of *FMR1* did not have any effect on *FMR4* expression, and *vice versa*. Interestingly, expression of both *FMR1* and *FMR4* is silenced in FXS patients. siRNA-mediated knockdown of *FMR4* followed by FACS analysis suggests the antiapoptotic function of *FMR4*. It should be noted that *FMR4* seems to be conserved in primates [43].

### *PRINS*

Another interesting lncRNA relevant to disease is *psoriasis susceptibility-related RNA gene induced by stress* (*PRINS*) that was identified a decade ago [44]. Psoriasis is a human-specific autoimmune skin disease affecting 2%−4% population, which has not yet been identified in other primates [45]. In psoriatic non-lesional epidermis, a 3.6-kb-long non-coding transcript was identified with two exons harboring three Alu elements collectively [44]. *PRINS* lncRNA is found to be specific to primates as well [45].

## Evolution of miRNAs

Evolutionary history of miRNAs is complicated. Although some miRNAs show deep sequence conservation throughout the animal kingdom, frequent gain and loss of miRNAs have also been observed [25], [46], [47]. This is because evolution of new miRNA genes, maybe due to their ability to form non-perfect fold back structures recognizable by miRNA machinery, is more likely to occur as compared to protein-coding genes [48]. There can be different ways by which a novel miRNA gene can arise in the genome. One way is the local or tandem duplication of an existing miRNA gene followed by subfunctionalization and neofunctionalization process [49], [50]. RNA molecules have the inherent property to form secondary structures. Gradual evolution of unstructured transcripts, often arising from introns to form hairpin structures identifiable by miRNA biogenesis machinery, can also give birth to new miRNA genes [48]. It has been observed that evolutionarily younger, species-specific miRNAs are more often located in introns [51]. These new miRNA genes are also enriched with TEs, since TEs can provide novel transcriptional units for the evolution of miRNA-like hairpins into novel miRNA genes [49], [52], [53], [54]. Another way by which novel miRNA genes can originate in the genome is through antisense transcription of an existing miRNA gene, resulting in the production of mature miRNA with a distinct seed sequence [55].

Comparative genomics studies indicate that primate-specific miRNAs contribute considerably to human-specific miRNAs. In one study, phylogenetic analysis of human miRNAs against metazoan genomes reveals that 41% of miRNAs originated in primates, with only 1% to be human-specific [46]. In another study, out of 1433 human miRNAs analyzed, 53% of them originated within great ape lineage, whereas 28% originated in hominoid lineage, and another 28% were associated with radiation of placental mammals. No more than 15% human miRNAs were conserved beyond placental mammals [47]. In a more recent study, 3707 novel miRNAs were found in the human genome, with 57% of them specific to humans [56]. Thus, the number of primate- or even human-specific miRNAs is large. Due to space limitation, we have not mentioned primate-specific miRNAs that are involved in cancer progression [22] or other cellular functions [57], [58].

### C19MC cluster

Although miRNA genes are often located within the introns of protein-coding genes, ‘free’ miRNA genes may also exist. While some miRNA genes can be solitary, they can also be organized in the form of clusters, suggesting origin from a common ancestor [18]. One such cluster known as chromosome 19 miRNA cluster (C19MC) is the largest one in human genome [59]. An exceptionally high gene density distinguishes chromosome 19 from other chromosomes [60]. With genomic span of 100 kb, C19MC consists of 46 genes encoding 59 different mature miRNAs [59]. Alu elements, which are spread over whole clusters, are thought to have facilitated the expansion of C19MC [61], [62]. Interestingly, when compared to other genomes, C19MC was exclusively present in primates. Despite its enormous size, the functions of miRNAs originating from this cluster are largely unknown. About 13.4% of the miRNAs expressed at early development are encoded by C19MC cluster [63], [64], [65], whereas their expression is non-significant in adult tissues [66], [67], suggesting a critical role of these miRNAs in embryogenesis. Intriguingly, C19MC is regulated by genomic imprinting. It is expressed exclusively from the paternal allele during early embryogenesis and become silent later on during development [68]. Notably, placenta is the only organ that escapes epigenetic silencing, where paternal allele remains active until birth [69].

**Figure 2:**
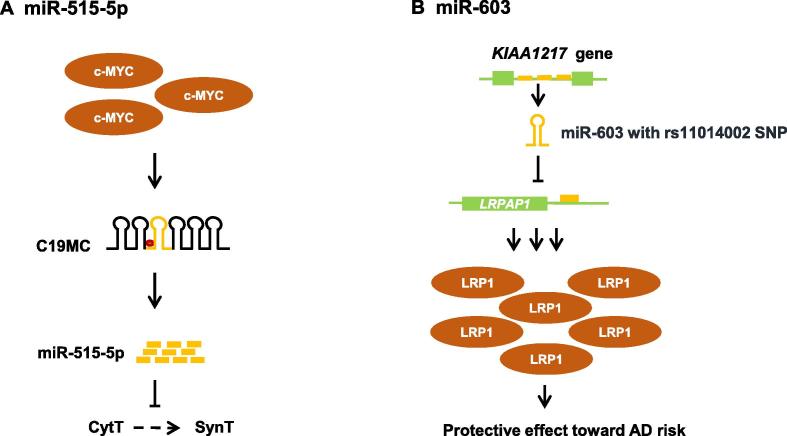
**Functional mechanisms of some primate-specific miRNAs** **A.** miR-515-5p resides in the C19MC. In preeclampsia, proto-oncogene c-MYC interacts with the E-boxes upstream of pri-miR-515. This interaction increases the expression of miR-515 thus inhibiting the differentiation of cytotrophoblasts (CytT) to syncytiotrophoblast (SynT). **B.** miR-603 resides in the intron of *KIAA1217* gene. Hair-pin structure of pre-miR-603 harboring rs11014002 SNP is stable. By directly targeting *LRPAP1*, miR-603 indirectly increases the expression of LRP1 protein, thus reducing the risk of AD. C19MC, chromosome 19 miRNA cluster; LRP1, low-density lipoprotein receptor-related protein 1; LRPAP1, LRP-associated protein 1; AD, Alzheimer’s disease.

Preeclampsia (PE) is a human pregnancy-specific disease, resulting from dysregulation of human trophoblast invasion and differentiation on or after the 20th gestation week. With significant maternal and neonatal morbidity and mortality, PE accounts for approximately 50,000 deaths worldwide annually [70], [71]. Upon differentiation of cytotrophoblast (CytT) to syncytiotrophoblast (SynT), there is a downregulation in expression of miR-515 family members that belong to C19MC. However, expression of miR-515-5p is markedly upregulated in the placenta of PE women [71]. This upregulation is owing to the interaction of the proto-oncogene protein c-MYC with the E-boxes upstream of pri-miR-515-1 and pri-miR-515-2, thereby inhibiting SynT differentiation (Figure2A). miR-515-5p target genes are critical for SynT differentiation. Their expression was decreased upon overexpression of miR-515-5p, thereby impairing SynT differentiation. This suggests that miR-515-5p plays a pivotal role in human trophoblast differentiation [71].

### X-linked primate-specific miRNA cluster

Another interesting primate-specific miRNA cluster is located at Xq27.3 of primate X-chromosome. The cluster is well conserved within primate genome, but is virtually absent from other mammalian genomes [72]. Absence of this cluster in non-primate genomes suggests that the formation of this cluster took place after primate-rodent split but before emergence of new-world monkeys. The cluster consists of 6 distinct miRNAs, which span a genomic region of approximately 33 kb. Functional analysis of members of this cluster shows that they are predominantly expressed in the human epididymis [72]. On the basis of their location and sequence similarity, members of this miRNA cluster can be phylogenetically divided into two regions, and each region is shaped by different evolutionary events. Among them, miR-890, miR-888, miR-892a, and miR-892b are tightly clustered within a genomic region of 3 kb and are highly similar with each other, suggesting evolution of these members as a result of tandem duplication events. The other two miRNAs (miR-891b and miR-891a), although similar to each other, do not show similarity with the other members of this cluster, suggesting that these two miRNAs may have experienced different evolutionary events to position them onto this locus [67], [72], [73]. *In silico* studies show that members of this miRNA cluster target genes controlling epididymal physiology, sperm maturity, male fertility, and tube development. Nonetheless, functional role of this miRNA cluster still needs to be further validated [72], [74].

### Neuropsychiatric disorder-related miRNAs

Among the neuropsychiatric disorders, major depressive disorder (MDD) is a prevalent mood disorder commonly treated using antidepressants [75], [76]. Metabolic glutamate receptor-4 (GRM4) modulates neurotransmitters, and is localized pre- and post-synaptically. Known for its involvement in regulation of anxiety-related behaviors, GRM4 is considered to be an attractive drug target [77], [78]. The primate-specific miR-1202 has recently been shown to target *GRM4* [79]. *In silico* sequence comparison among 100 animal genomes shows that miR-1202 is present only in primates. miR-1202 is further validated experimentally to have higher expression in humans as compared to non-human primates [79]. Notably, higher GRM4 protein expression is also detected in postmortem brain samples and clinical samples from patients with depression, suggesting negative correlation with the expression of miR-1202 [79]. These studies suggest that miR-1202 could be a potential target for new antidepressant treatment as well as biomarker for treatment prediction and response [79]. Bipolar disorder (BD) is another neuropsychiatric disorder characterized by frequent mood swings between depression and mania. Abnormalities in neuronal synapses are thought to be a contributing factor toward pathogenesis of BD [80]. For instance, a recent study has shown the association between a primate-specific miRNA, miR-1908-5p, and the pathogenesis of BD. Previously uncharacterized, miR-1908-5p is shown to target genes that function in neuronal glutamatergic synapses, which includes *DLGAP4*, *GRIN1*, *STX1A*, *CLSTN1*, and *GRM4*. *In silico* brain expression profiles also show inverse correlation between the expression of miR-1908-5p and its target genes [80].

### Alzheimer’s disease-related miRNAs

Alzheimer’s disease (AD) is a neurodegenerative disorder leading to progressive loss of memory, behavioral issues and functional decline in ability to learn. It accounts for more than 80% of the dementia cases globally [81]. Low-density lipoprotein receptor-related protein 1 (LRP1) is an important player in preventing neurodegeneration. LRP1 maintains brain lipid homeostasis in order to maintain neuronal integrity [82]. Patients with AD have reduced protein levels of LRP1 in middle frontal cortexes, whereas LRP1 level in unaffected individuals negatively correlates with the age [82]. Primate-specific miR-603 has recently been shown to have potential role in the pathogenesis of AD. miR-603 is an intronic miRNA expressed from the host gene *KIAA1217*, and is highly expressed in brain [83]. Interestingly, a single nucleotide polymorphism (SNP), rs11014002, in the pre-miR-603 increase stability of the hairpin structure and the production of mature miRNA. LRP-associated protein 1 (LRPAP1) controls LRP1 protein expression by acting as a molecular chaperone to LRP1 [83]. By directly targeting *LRPAP1* mRNA, miR-603 indirectly increases the levels of LRP1 protein (Figure2B). Taken together, these observations suggest a potential protective role of miR-603 against AD [84].

## Conclusion and perspective

Human genome is estimated to have approximately 20,000 protein-coding genes, which account for less than 2% of the human genome. Could the number of protein-coding genes be correlated to the organismal complexity? Less complex eukaryotes such as nematode *Caenorhabditis elegans* contain a similar number of protein-coding genes as humans, and about 70% proteins in *C. elegans* have homologs in humans [85]. It is increasingly appreciated that organismal complexity is not solely dependent on number of protein-coding genes. Alternative splicing of pre-mRNAs as well as modifications on proteins in order to increase diversity and functionality of the proteome play some part in the complexity of an organism as well. Since the identification of lncRNA *H19*, which is induced during liver development in mice [86], [87], and miRNA lin-4, which regulates LIN-14 in *C. elegans* [88], there has been a constant increase in the repertoire of ncRNAs owing to the development in RNA sequencing technologies and bioinformatics. Till now, thousands of lncRNAs and miRNAs have been identified, however, the function of many of them have not been experimentally shown yet. Lines of compelling evidence support that ncRNAs are one of, if not the major, drivers of evolution. It is highly possible to detect lineage-specific miRNAs with deep sequencing technology due to quick turnover rate of miRNAs and target sites [89]. It is, therefore, important to differentiate miRNAs detected through RNA-sequencing from experimentally verified functional lineage-specific miRNAs. In this review, we have compiled the primate-specific lncRNAs and miRNAs that have been verified experimentally (Table 1). Beside lncRNAs and miRNAs, we also need to keep in mind that there are other ncRNAs such as circular RNAs that may have primate- or human-specific members [90], [91]. The lncRNAs and miRNAs we discussed are associated with diverse functions, ranging from alternative splicing to neuro development, and some of them are involved in development or human diseases. Therefore, primate-specific ncRNAs can be important diagnostic or therapeutic targets.

**Table 1:** **List of primate-specific lncRNAs and miRNAs discussed in this review**

**Category**	**Name**	**Functional role**	**Refs.**
lncRNA	*5S-OT*	Alternative splicing	[37]
	*lncND*	Neuro development	[39]
	*lincHPAT*	Embryo development	[40]
	*FMR4*	Antiapoptotic	[43]
	*PRINS*	Psoriasis disease	[44]

miRNA	C19MC	Preeclampsia; cancers	[68]
	X-linked miRNA cluster	Possible roles in epididymal physiology; sperm maturity; male fertility; tube development	[73], [74]
	miR-1202	Major depressive disorder	[79]
	miR-1908-5p	Bipolar disorder	[80]
	miR-603	Pre-miR-603 with rs11014002 SNP having protective effect toward Alzheimer’s disease	[83], [84]

Despite the great advances, there are some limitations that need to be overcome for future studies. For example, one limitation in functional studies of primate-specific ncRNAs is the lack of model organism for *in vivo* studies. In cancer research, mouse model is an important tool, including germline manipulation and xenograft of human tumors. Exposure of mice to potential carcinogens such as pharmaceutical products, industrial or agricultural agents is a standard method in evaluating hazards of these agents exposing to human population [92]. However, there are examples of rodent carcinogens that are not carcinogenic in humans such as anticonvulsant drug phenobarbital [93]. This shows that although there is a fundamental similarity of carcinogenesis, yet differences in the cancer biology between species do exist [94], [95]. To understand roles of disease-related primate-specific ncRNAs, availability of animal models is essential. For instance, use of monkeys in research can be one option as miRNA dysregulation has been studied in the Huntington’s disease monkey, a transgenic primate model of a human disease [96].

Evolutionarily young ncRNAs hold immense potential in therapeutics where they can serve as novel biomarkers and/or therapeutic targets, especially in diseases affecting organs that have been restructured during primates and human evolution. Without a doubt, future investigations in primate- and human-specific ncRNAs would deepen our understanding of distinctions between primates and other mammals, and may provide medical benefits to humans.

## Competing interests

The authors declare no competing interests.
